# Use of Contrast-Enhanced Ultrasound with Sonazoid for Evaluating the Radiotherapy Efficacy for Hepatocellular Carcinoma

**DOI:** 10.3390/diagnostics11030486

**Published:** 2021-03-09

**Authors:** Akihiro Funaoka, Kazushi Numata, Atsuya Takeda, Yusuke Saigusa, Yuichirou Tsurugai, Hiromi Nihonmatsu, Makoto Chuma, Hiroyuki Fukuda, Masahiro Okada, Masayuki Nakano, Shin Maeda

**Affiliations:** 1 Gastroenterological Center, Yokohama City University Medical Center, Yokohama, Kanagawa 232-0024, Japan; afunaoka@yokohama-cu.ac.jp (A.F.); h.n.twopines@gmail.com (H.N.); chuma@yokohama-cu.ac.jp (M.C.); fukuhiro@yokohama-cu.ac.jp (H.F.); 2 Division of Gastroenterology, Yokohama City University Graduate School of Medicine, Yokohama, Kanagawa 236-0004, Japan; smaeda@med.yokohama-cu.ac.jp; 3 Radiation Oncology Center, Ofuna Chuo Hospital, Kamakura, Kanagawa 247-0056, Japan; takeda@1994.jukuin.keio.ac.jp (A.T.); tatesen2000@gmail.com (Y.T.); 4 Department of Biostatistics, Yokohama City University Graduate School of Medicine, Yokohama, Kanagawa 236-0004, Japan; saigusay@yokohama-cu.ac.jp; 5 Department of Radiology, Nihon University School of Medicine, Tokyo 173-8610, Japan; okada.masahiro@nihon-u.ac.jp; 6 Tokyo Central Pathology Laboratory, Hachioji, Tokyo 192-0024, Japan; masayukinakano23@gmail.com

**Keywords:** hepatocellular carcinoma, radiotherapy, contrast-enhanced ultrasound, sonazoid

## Abstract

Radiotherapy is one of the available curative therapies for hepatocellular carcinoma (HCC). We investigate the use of contrast-enhanced ultrasound using Sonazoid (SCEUS) in evaluating the efficacy of radiotherapy for HCC. We enrolled 59 patients with 59 HCCs in this retrospective study. Tumor size and tumor vascularity were evaluated using SCEUS before and 1, 3, 7, 10, and 13 months after radiotherapy. The median follow-up period was 44.5 months (range: 16–82 months). Of the HCCs, 95% (56/59) had no local recurrence, while 5% (3/59) did. At 13 months after radiotherapy, in cases with no local recurrence, SCEUS showed a reduction in tumor vascularity in all cases, while tumor size reduction (>30% reduction, compared with pre-radiotherapy) was observed in 82.1% (46/56). In all three cases of local recurrence, vascularity and tumor size reduction were not observed during the follow-up period and residual HCCs were demonstrated pathologically. Compared with cases with local recurrence, tumor size reduction and reduction in tumor vascularity (*p* < 0.001) were significantly greater in cases with no local recurrence at 13 months after radiotherapy. SCEUS may be useful in evaluating radiotherapy efficacy for HCC.

## 1. Introduction

The global incidence of primary liver cancer is 841,080 people, accounting for 4.7% of all cancers [1,2]. Hepatocellular carcinoma (HCC) accounts for 75 to 85% of all primary liver cancers [1], and HCC incidence continues to increase [3]. The HCC treatment options vary, depending on the prognostic stage; surgical resection, liver transplantation, and radiofrequency ablation (RFA) are the only curative treatments [4,5,6]. Recently, radiotherapy (RT), such as stereotactic body radiotherapy (SBRT), has become widely used in clinical practice as a second-line treatment option, especially in patients ineligible for surgical resection or liver transplantation, or those for whom loco-regional therapies, such as RFA or transarterial chemoembolization, have failed [7,8,9]. RT has excellent local control and overall survival in patients with good liver function and can compensate for the shortcomings of other treatments [10,11]. RT appears to be an acceptable alternative treatment option for patients who are not candidates for RFA [10,11].

In general, contrast-enhanced computed tomography (CECT) and contrast-enhanced magnetic resonance imaging (CEMRI) are recommended for follow-up after RT to evaluate treatment response and for long-term follow-up in short intervals (usually 3–6 months in the first year) [12,13,14,15,16,17,18,19,20,21]. According to the several reports on CECT and CEMRI, the accurate assessment of the efficacy of RT is sometimes difficult, as the surrounding liver parenchyma around the HCC shows hypervascularity for a long time after RT. This finding masks the accurate evaluation of the vascularity of the HCC [13,14,15,16]. In cases of patients with chronic kidney disease or allergy to contrast agents, these imaging modalities cannot be used. In this situation, contrast-enhanced ultrasound (CEUS) is the most appropriate diagnostic imaging modality, as it does not produce nephrotoxic or hepatotoxic effects due to the contrast agent [22]. In addition, unlike CECT and CEMRI, CEUS can be used to observe hemodynamics changes in the HCC and the surrounding liver parenchyma, in real-time, after injection of the ultrasound contrast agent. Another advantage of CEUS is that it is repeatable. Additional injections of the ultrasound contrast agent are useful for the accurate assessment of tumor vascularity.

The European Federation of Societies for Ultrasound in Medicine and Biology guidelines highlight the role of CEUS as a cost-effective technique with a good safety profile, not only for the characterization and detection of focal liver lesions, but also for monitoring tumor response after curative, loco-regional, or systemic treatment for HCC [23,24,25]. CEUS is useful for assessing tumor vascularity, based on previous studies [26,27]. Perflubutane-based microbubble contrast agent (Sonazoid^®^, Daiichi Sankyo, Tokyo, Japan) has been widely used in clinical practice in Japan, South Korea, Taiwan, and Norway.

In the present study, we investigate the use of CEUS with Sonazoid (SCEUS) for evaluating RT efficacy for HCCs with a follow-up period of at least 13 months.

## 2. Materials and Methods

### 2.1. Patient Enrollment

Between April 2013 and July 2018, 63 consecutive patients with 63 HCCs belonging to our institution were treated by RT at the Radiation Therapy Center at Ofuna Chuo Hospital, Kamakura, Japan. All SCEUS procedures were performed at our institution. Four HCCs were excluded, for the following reasons: Deep location in the liver and could not be assessed by grayscale ultrasound (US) or SCEUS (n = 3), or location near the diaphragm and could not be assessed by grayscale US and SCEUS (n = 1). Finally, 59 patients with 59 HCCs were enrolled in this retrospective study. This study was conducted in accordance with the Declaration of Helsinki. Institutional review board approval and informed consent from all patients were obtained for this retrospective study (number B180900067).

The inclusion criteria were as follows: (1) Unresectable HCC due to infeasibility, difficulty, or patient refusal; (2) unsuitable for RFA, due to adjacency to the portal vein, hepatic vein, diaphragm, or heart, or patients who had a hemorrhagic tendency; (3) single HCC in patients at stage 0,A in Barcelona Clinic Liver Cancer (BCLC) staging; (4) patients with HCC detectable in grayscale US before RT; (5) patients who can sufficiently hold their breath when performing SCEUS; (6) Child–Pugh A chronic hepatitis, cirrhosis, or Child–Pugh B (Child–Pugh score 7) cirrhosis; and (7) ability to follow-up for more than 13 months after RT. The exclusion criteria were uncontrolled ascites or extrahepatic lesions.

### 2.2. Imaging Method

#### 2.2.1. US Imaging

We assessed the detection of HCC lesions using the LOGIQ E9 ultrasound system (GE Healthcare, Ltd., Chicago, IL, USA) with native tissue harmonic grayscale imaging using a convex probe with a frequency of 1–6 MHz and a micro-convex probe with a frequency of 2–5 MHz (hereafter referred to as grayscale US).

#### 2.2.2. SCEUS Procedures

As previously reported [28,29], a 0.2 mL dose of Sonazoid was injected into a forearm cutaneous vein at 0.2 mL/s using a 24-gauge cannula, followed by 2 mL of 5% glucose after the Sonazoid injection. SCEUS images were acquired during three contrast phases, consisting of an arterial phase (AP; 10–50 s after initiation of injection), a portal phase (80–120 s after initiation of injection), and a PVP (10 min after initiation of injection). Since 2014, grayscale US images obtained with the newly developed transducer have been used for native tissue harmonic imaging, which uses wide-band phase inversion harmonic grayscale imaging and software to reduce speckles. This technique provides a high spatial resolution and deep penetration with high frame rates (28–30 frames per second). Using SCEUS based on native tissue harmonic imaging at a low mechanical index (MI; 0.28) and high frame rate (28–30 frames per second), tumor vessels and tumor stains can be evaluated in detail and in real-time [28,29]. In cases of hyperechoic lesions, high MI (0.7–1.2) contrast imaging was used to accurately evaluate the presence or absence of defective areas during the PVP [30].

#### 2.2.3. CT Imaging

CECT was performed using a 16 multi-detector CT scanner (Aquilion 16; Canon Medical, Ootawara, Japan) with a tube voltage of 120 kV, a tube current setting at the automatic milliampere exposure setting, a reconstruction section and interval thickness of 5 mm, a pitch of 15, and a gantry speed of 0.5 s per rotation. A nonionic contrast agent, iopamidol (Iopamiron 300 or 370; Bayer Schering Pharma AG, Berlin, Germany), was injected. Then, a power injector (Dual Shot GX; Nemoto Kyorindo, Tokyo, Japan) was used to inject 100 mL of iopamidol at 3 mL/s through a catheter placed in the antecubital vein. The scanning time in the arterial phase was confirmed using an automatic bolus-tracking program (RealPrep, Canon Medical, Ootawara, Japan). The trigger point for starting AP scanning was set to an attenuation of 230 HU from the baseline attenuation of the abdominal aorta. Portal phase scanning was performed 70 s after contrast injection, and equilibrium phase images were acquired 180 s after injection.

#### 2.2.4. MR Imaging

MR imaging was performed using a 1.5 T whole-body imager (Avant; Siemens Medical System, Erlangen, Germany). A power injector (Spectris Solaris EP; MEDRAD, Bayer Schering Pharma AG, Berlin, Germany) was used to inject 0.1 mL/kg of gadolinium-ethoxybenzyl-diethylenetriamine pentaacetic acid (Gd-EOB-DTPA; Primovist; Bayer Schering Pharma AG, Berlin, Germany) at 1 mL/s through a catheter placed in the antecubital vein, followed by flushing with 20 mL of sterile saline solution at 2 mL/s. Arterial phase, portal phase, and late phase scanning were performed at 25–30, 70–85, and 180 s, respectively, after initiation of the contrast injection, while hepatobiliary phase scanning was performed 20 min after initiation of the contrast injection.

### 2.3. Radiotherapy (RT)

As previously described [31], three kinds of risk-adapted dose prescriptions were used: SBRT, with total doses of 35 Gy or 40 Gy, were delivered in five fractions over 5–7 days (depending on liver function), such that the dose to a 10-cc area of the gastrointestinal tract was limited to <25 Gy. A total dose of 35 Gy was administered to patients with Child–Pugh class B or A disease, with >20% of the normal liver receiving >20 Gy, and a total dose of 40 Gy in the other patients. When exceeding the limitation of the gastrointestinal tract, hypofractionated radiotherapy (HFRT), with a total dose of 36–45 Gy, was delivered in 12–15 fractions over 16–21 days. The treatment was planned to enclose the planning target volume with a 60–80% isodose line of the maximal dose.

### 2.4. Evaluation of the Therapeutic Efficacy of RT by SCEUS

All 59 cases were evaluated at 1, 4, 7, 10, and 13 months after RT treatment. Follow-up with imaging assessments every three months was continued as long as the patient was alive and could visit the hospital. The examination interval between CECT/CEMRI and SCEUS was about one week. Using the US procedure, the following were evaluated: (1) The change in tumor size using grayscale US or re-injection of Sonazoid in the PVP SCEUS, (2) tumor vascularity in AP or re-injection during PVP SCEUS, (3) hypervascularity of surrounding liver parenchyma during AP SCEUS, and (4) both irradiated HCC and surrounding liver parenchyma becoming a hypoechoic area (perfusion defect) in PVP SCEUS. A tumor size reduction was defined as a reduction of more than 30%, compared to the maximum diameter of the HCC lesion measured before RT treatment with grayscale US. An increase in tumor size was defined as an increase of more than 20% and more than 5 mm in diameter, compared to the maximum diameter of the HCC lesion measured before treatment with grayscale US. If a HCC lesion was unclear on grayscale US, but the HCC lesion and the surrounding liver parenchyma showed a hypoechoic area (perfusion defect) during PVP SCEUS, additional Sonazoid re-injection was performed during PVP, in order to evaluate the vascularity of the target HCC lesion and surrounding liver parenchyma. If the target HCC showed hypovascularity, compared with the surrounding liver parenchyma, and appeared as a perfusion defect during AP of re-injection, the target lesion was detected and measured. Local recurrence was suspected if the target HCC showed hypervascularity during AP SCEUS and a perfusion defect during PVP. Additional injections of Sonazoid during PVP were used to determine whether or not arterial blood flow entered the perfusion defect (defect re-injection method). If the fast wash-in of arterial flow was observed in the perfusion defect, this was considered a confirmatory sign of local recurrence [32]. All SCEUS was performed independently by two different experienced radiologists (H.N. and A.F.), who were blind to clinical information of the patients. The results of the SCEUS and grayscale US were evaluated by an expert hepatologist (K.N.) with 20 years of experience in HCC diagnosis and treatment.

### 2.5. Evaluation of the Therapeutic Efficacy of RT by CECT/CEMRI

Of the 59 patients, 54 were available for contrast agents and were evaluated by CECT or CEMRI, performed 1, 4, 7, 10, and at least 13 months after RT. The presence or absence of local recurrence was assessed according to the evaluation of vascularity of the target HCC lesion and its size. Local recurrence was suspected if the target HCC showed hypervascularity during the AP and wash-out during the subsequent phases without tumor size reduction. For the remaining five patients, who had renal dysfunction or contrast allergy, non-enhanced CT and non-enhanced MRI were performed. In those cases, changes in tumor size were evaluated in non-enhanced CT or MRI; in non-enhanced MRI, the presence or absence of high signal intensity in the diffusion-weighted image (DWI) was observed.

Follow-up with imaging assessments every three months was continued as long as the patient was alive and could visit the hospital. To evaluate the effects of RT, the CECT/CEMRI findings obtained before and after the RT were evaluated by two hepatologists (M.C. and H.F.), neither of whom was involved in the RT treatment, and both of whom were blind to the clinical information and other radiological findings of the patients. Each of the hepatologists had 20 years of clinical experience in evaluating MRI.

### 2.6. Follow-Up of Alpha-Fetoprotein (AFP)

Blood tests for AFP level were performed and followed up in the same way as for the imaging studies. AFP level was considered abnormal at >10 ng/mL.

### 2.7. Diagnosis of Presence or Absence of Local Recurrence

If neither reduction in tumor vascularity nor tumor size were observed by imaging modalities, as mentioned above, local recurrence was suspected. Tumor biopsy was performed under US guidance and the pathological results were used as the gold standard for the presence of local recurrence. In these cases, additional treatment was performed.

If reductions in either tumor vascularity or tumor size were observed by the imaging modalities, no local recurrence was suspected. If these findings (as mentioned above) were observed on the basis of the imaging examinations at 16 months or more of follow-up, the patient was considered to have no local recurrence. Therefore, the gold standard for determining no local recurrence was imaging modalities alone.

The follow-up period for the diagnosis of presence or absence of local recurrence ended on 31 August 2020.

### 2.8. Statistical Analysis

We compared and examined the SCEUS findings between cases with and without local recurrence. To determine whether AFP was effective in follow-up after RT, Fisher’s exact test was performed for cases with and without local recurrence in which the AFP was originally high. The SCEUS findings observed in the cases were examined using a Fisher’s exact test, in order to determine whether significant differences existed between cases with and without local recurrence. Statistical analysis was performed to calculate the sensitivity, specificity, and accuracy of the findings of the tumor size or vascularity for the diagnosis of local recurrence after RT. *p* < 0.05 was considered to be statistically significant. All statistical analyses were performed using the IBM SPSS statistics version 26.0 software (IBM SPSS, Chicago, IL, USA).

## 3. Results

### 3.1. Patient Characteristics

Table 1 shows the clinical characteristics of the enrolled patients who were treated using RT and followed up by SCEUS. A total of 59 cases with 59 HCC lesions were eligible for inclusion in this study. SBRT was administered to 48 cases and HFRT was administered to 11. The median tumor size was 19.6 mm (Table 1). RT was performed due to anticoagulation in 4 cases, adjacent to the dome in 23 cases, posterior to the portal vein in 23 cases, lesions in segment 1 of the liver in 6 cases, lesions in segment 7 of the liver in 1 case (i.e., lesions just below the diaphragm), and adjacent to the intestinal organs and heart in 2 cases.

### 3.2. Achievement of Each Imaging Modality

**Figure 1:**
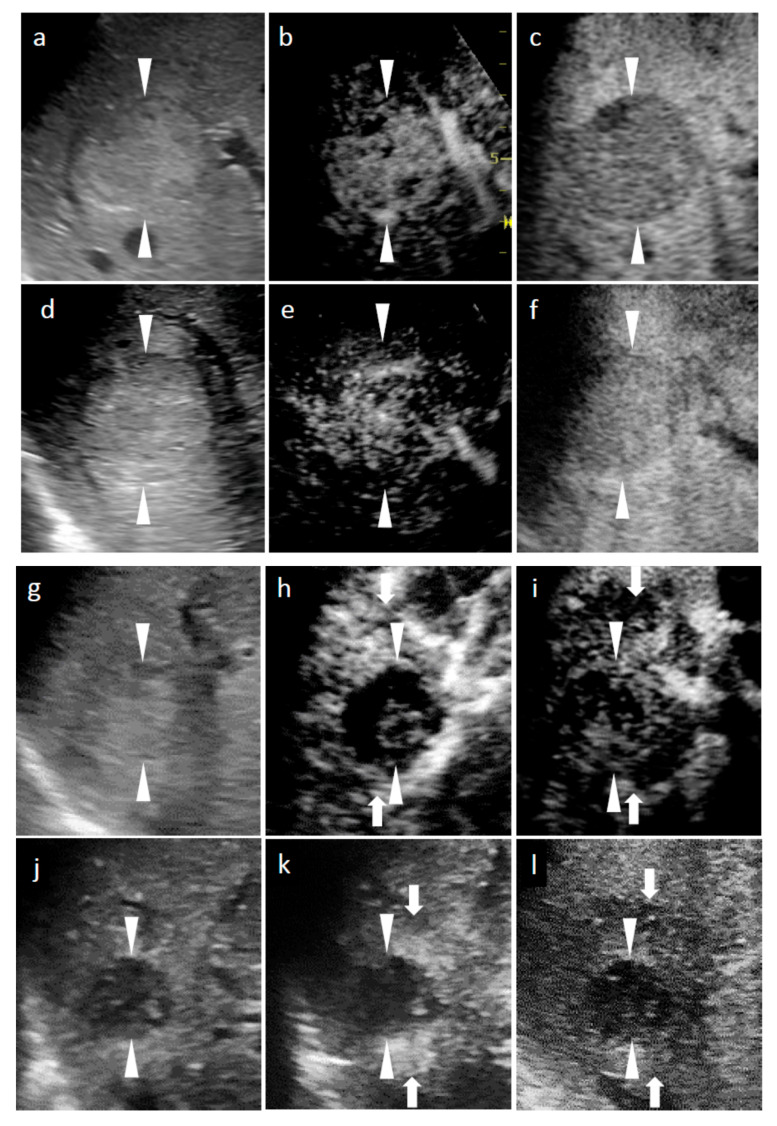
Grayscale ultrasound (US) and contrast-enhanced US with Sonazoid (SCEUS) images, before and after radiotherapy (RT), for a hepatocellular carcinoma (HCC) lesion (maximum diameter: 38 mm) in segment VIII; evaluated as no local recurrence after RT. The patient was unable to undergo punctuation or surgical resection as they took two antiplatelet drugs during the subsequent 6 months of coronary stenting due to an angina attack. After RT, the patient could not receive contrast-enhanced computed tomography (CT), due to progression of chronic kidney disease. (**a**–**c**) Before RT: A hyperechoic lesion was observed using grayscale US (**a**). This lesion showed hypervascularity during the arterial phase (AP) of SCEUS (**b**) and hypoechoic (perfusion defect) during the post-vascular phase (PVP) (**c**). One month after RT (**d**–**f**): Grayscale US showed a hyperechoic nodule (**d**). This lesion showed hypervascularity during AP SCEUS (**e**) and appeared hypoechoic during the PVP (**f**) one month after RT. Grayscale US and SCEUS findings obtained after RT showed no remarkable changes, compared with those obtained before RT. Four months after RT (**g**–**i**): Grayscale US showed a hyperechoic nodule and a reduction in tumor size (**g**). AP SCEUS showed decreased vascularity of the HCC lesion and hypervascularity of the surrounding liver parenchyma during the arterial phase (**h**). Both the HCC lesion and the surrounding liver parenchyma appeared as a perfusion defect during the post-vascular phase (**i**). Thirteen months after RT (**j**–**l**): Grayscale US showed a hypoechoic nodule and a marked reduction in tumor size (**j**). AP SCEUS showed a disappearance of vascularity of the HCC lesion and slight hypervascularity of the surrounding liver parenchyma (**k**). Both the HCC lesion and the surrounding liver parenchyma appeared as perfusion defect during the PVP (**l**). Compared with four months after RT, the size of perfusion defect decreased apparently. Arrowheads indicate the margins of the HCC lesion. Arrows show the margins of irradiated surrounding liver parenchyma.

All cases received SCEUS procedures during follow-up periods after RT. At the beginning of the study, four patients could not undergo CECT or CEMRI due to renal dysfunction (two cases; Figure 1), allergy to the contrast media (one case), and asthma (one case). At 10 months after RT, one case could not undergo CECT or CEMRI, due to appearance of an allergy to the contrast media. These five cases had no local recurrence.

### 3.3. Efficacy of RT

**Figure 2:**
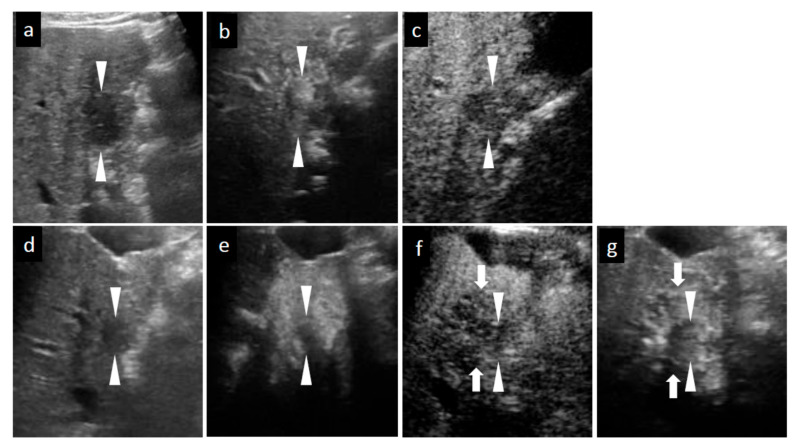

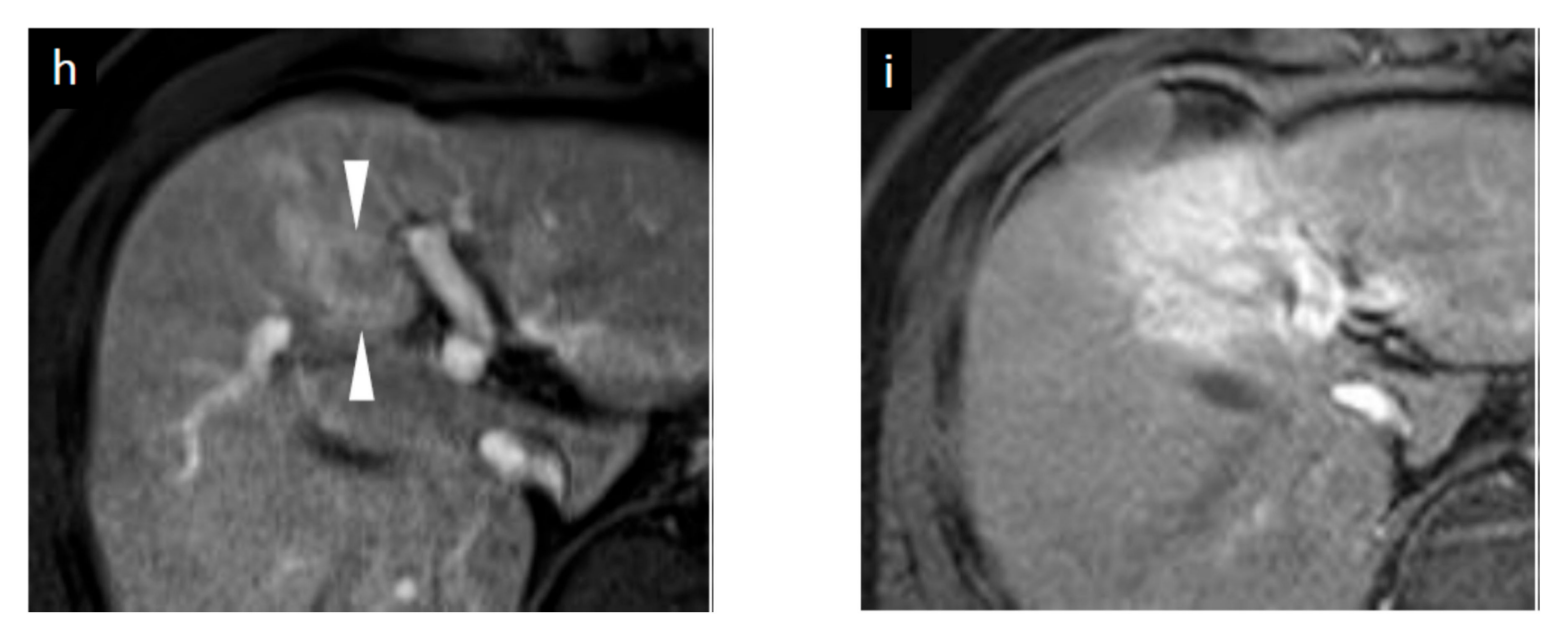
Grayscale ultrasound (US), contrast-enhanced US with Sonazoid (SCEUS), and contrast-enhanced MRI (CEMRI) images before and seven months after radiotherapy (RT) for a HCC lesion (maximum diameter: 22 mm) in segment IV, evaluated as no local recurrence after RT. A safe punctuation was difficult, as the lesion was located behind the gallbladder. The patient refused surgical resection, so they received RT. SCEUS before RT (**a**–**c**): A hypoechoic lesion was observed using grayscale US (**a**). This lesion showed hypervascularity during the arterial phase (AP) of SCEUS (**b**) and hypoechoic (perfusion defect) during the post vascular phase (PVP) (**c**). SCEUS seven months after RT (**d**–**g**): Grayscale US showed a hypoechoic nodule and a reduction in tumor size (**d**). AP SCEUS showed a disappearance of vascularity of the HCC lesion and hypervascularity of the surrounding liver parenchyma (**e**). Both the HCC lesion and the surrounding liver parenchyma appeared as a perfusion defect during the PVP (**f**). After re-injection of Sonazoid during the PVP, the HCC showed hypovascularity and the surrounding liver parenchyma showed hypervascularity (**g**). AP CEMRI before (**h**) and seven months (**i**) after RT: Before RT, this lesion showed hyperintensity during AP CEMRI (**h**). Both the HCC lesion and the surrounding liver parenchyma appeared as hyperintensity at seven months after RT (**i**). Seven months after RT, we observed a discrepancy between the vascularity shown by SCEUS and that by CEMRI, but the lesion has not recurred for more than 3 years, suggesting that the SCEUS findings might be correct. Arrowheads show the margins of the HCC lesion. Arrows show the margins of irradiated surrounding liver parenchyma.

**Figure 3:**
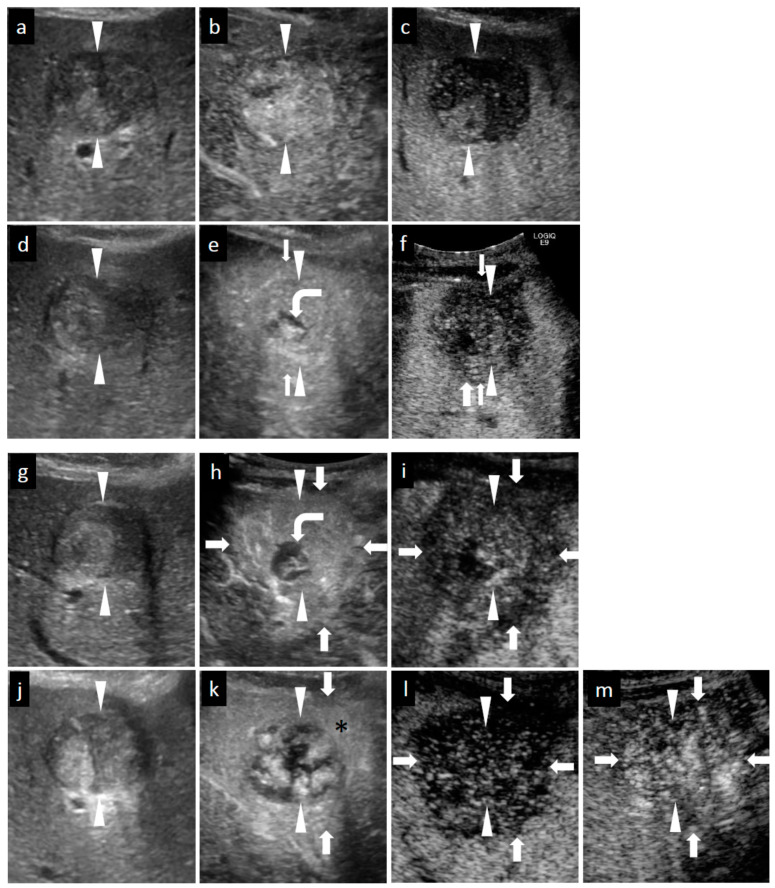

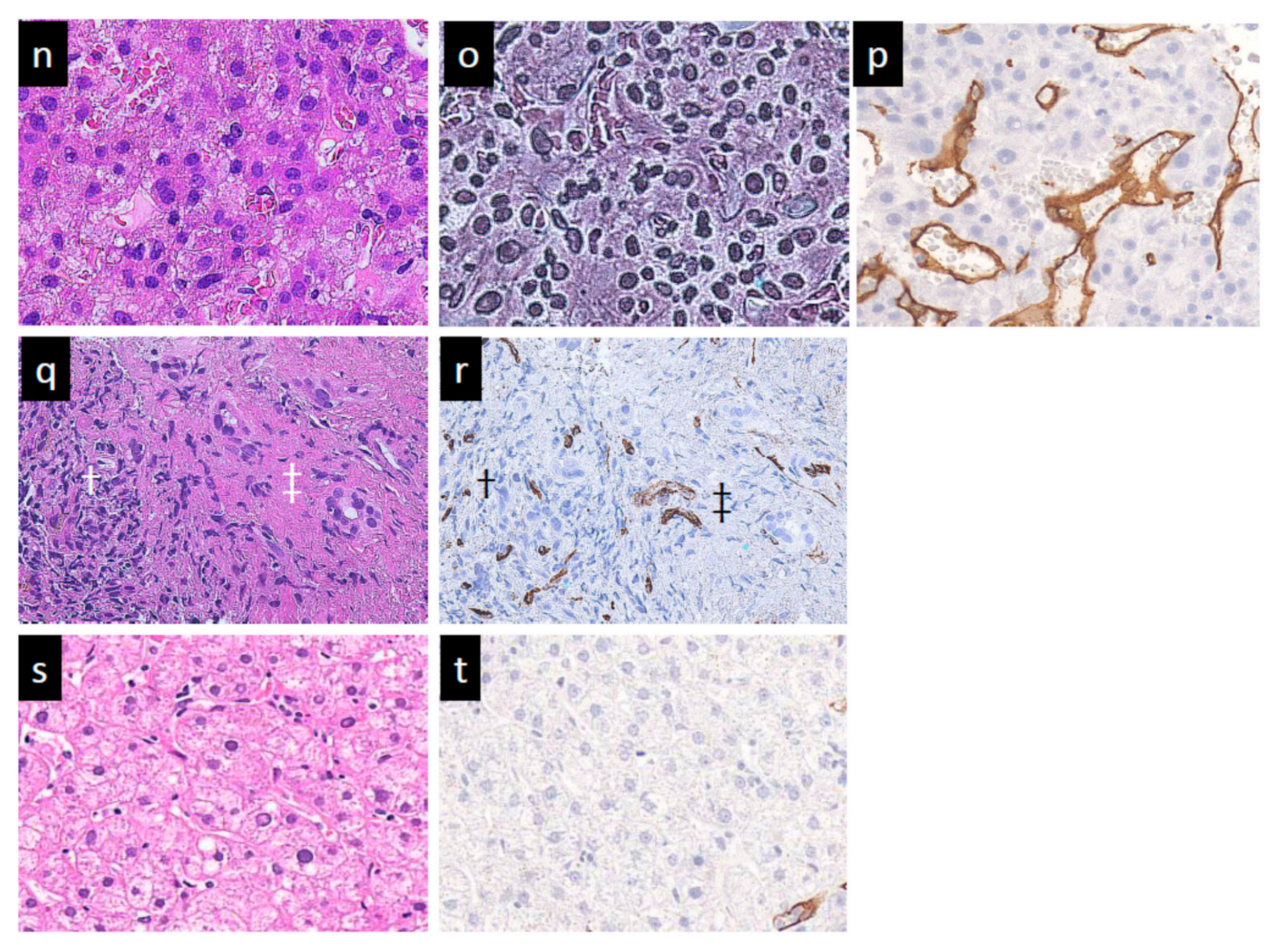
Grayscale ultrasound (US) and contrast-enhanced US with Sonazoid (SCEUS) images before and after radiotherapy (RT) for an HCC lesion (maximum diameter: 34 mm) in segment III/IV, evaluated as residue after RT. RT was selected as treatment because the lesion was located adjacent to the left portal vein and the tumor size was more than 3 cm (as the maximum diameter). Hypofractionated radiotherapy was performed as the bowel organ was adjacent to the lesion. Before RT (**a**–**c**): A hyperechoic lesion was observed using grayscale US (**a**). This lesion showed hypervascularity during arterial phase (AP) SCEUS (**b**) and hypoechoic (perfusion defect) during the post-vascular phase (PVP) (**c**). One month after RT (**d**–**f**): The tumor size did not change on grayscale US (**d**). Using SCEUS, the HCC lesion showed hypervascularity with a partially hypovascular area (curved arrow) during the AP (**e**) and was hypoechoic during the PVP (**f**). The surrounding liver parenchyma showed slight hypervascularity during the AP (**e**) and was hypoechoic during the PVP (**f**). Four months after RT (**g**–**i**): The tumor size did not change on grayscale US (**g**). Using SCEUS, the HCC lesion showed hypervascularity with a partially hypovascular area (curved arrow) during the AP (**h**). The surrounding liver parenchyma showed apparent hypervascularity during the AP (**h**). Both the HCC lesion and the surrounding liver parenchyma appeared as a perfusion defect during the PVP (**i**). Thirteen months after RT (**j**–**m**): The tumor size did not change on grayscale US (**j**). Using SCEUS, compared with 4 months after RT, the HCC lesion showed hypervascularity during the AP (**k**). AP SCEUS showed slight hypervascularity of the surrounding liver parenchyma (**k**). Both the HCC lesion and the surrounding liver parenchyma appeared as a perfusion defect during the PVP (**l**). The boundary between the HCC and the surrounding liver parenchyma was unclear (**l**). After re-injection of Sonazoid during the PVP, both the HCC and the surrounding liver parenchyma showed hypervascularity (**m**). In this case, tumor size reduction and the disappearance of tumor vascularity were not observed during 13 months follow-up. Arrowheads indicate the margins of the HCC lesion. Arrows show the margins of the irradiated surrounding liver parenchyma. Pathological findings obtained from irradiated HCC area (**n**–**p**), irradiated surrounding non-tumor area (**q**,**r**), and non-irradiated non-tumor area (**s**,**t**) at 13 months after RT. For the irradiated HCC area, hematoxylin and eosin (HE) staining showed obvious cell atypia. Specifically, hypercellularity and larger, irregularly shaped nuclei can be seen. The nuclear cytoplasmic ratio is significantly higher. A few multinucleated giant cancer cells can be seen (**n**). Silver staining showed that reticular fibers have totally disappeared, resulting in poorly differentiated HCC (**o**). CD34 staining showed strong diffuse expression of CD34, suggesting increased neovascularization resulting from sinusoidal capillarization and the formation of sinusoid vascular endothelium in HCC (**p**). Irradiated surrounding non-tumor area showing hypervascularity during arterial phase SCEUS (* mark seen in (**k**)): HE staining showed neither cancer cells nor hepatocytes. Infiltration of inflammation cells (†) and severe fibrosis (‡) are noted (**q**). CD34 staining showed diffuse expression of CD34 in infiltration of inflammation cells (†) and severe fibrosis regions (‡) (**r**). Non-irradiated non-tumor area: HE staining showed normal hepatocytes with the normal nucleus shape and the nuclear cytoplasmic ratio. Thin trabeculae are clearly visualized (**s**). The negative expression of CD34 is seen (**t**).

During the follow-up period (median, 44.5 months; range, 16–82 months) after RT, 94.9% (56/59) of cases had no local recurrence (Figure 1 and Figure 2). In cases with no local recurrence, 47 patients underwent SBRT and 9 patients underwent HFRT. Follow-up examinations continued beyond 13 months after RT. No local recurrence was observed at 16 months or more after RT. The remaining 5.1% (3/59) of cases showed local recurrence and two of them underwent HFRT (Figure 3). These three cases with local recurrence were pathologically diagnosed as viable HCCs using ultrasound-guided biopsy. Two of the three cases were diagnosed as poorly differentiated HCCs (Figure 3n–t) and the remaining one was well-differentiated HCC.

### 3.4. Changes in SCEUS and CECT/CEMRI Findings during Follow-Up Periods

#### 3.4.1. SCEUS Findings of the Cases with No Local Recurrence

Table 2 summarizes the changes of tumor size and tumor vascularity in the SCEUS and CECT/CEMRI findings during the 13 months after RT for cases without local recurrence. This table also shows changes in SCEUS findings of the tumor and irradiated liver parenchyma surrounding the tumor after RT. At 1 month after RT, reductions in tumor vascularity and tumor size evaluated by SCEUS were observed in 21.4% and 7.1% of cases, respectively. At 4 months after RT, the AP SCEUS showed decreased vascularity of the irradiated HCC (87.5%) and increased vascularity of the irradiated surrounding liver parenchyma (89.3%). In the PVP, both the irradiated HCC and the surrounding liver parenchyma appeared as a hypoechoic area (perfusion defect; 87.5%; see Figure 1 and Figure 2). At 7 months after RT, in addition to the findings observed at 4 months after RT mentioned above, tumor size reduction was observed (62.5%). At 13 months after RT, tumor size reduction (82.1%) and decreased vascularity of the irradiated HCC (100%) were observed.

#### 3.4.2. SCEUS Findings of the Local Recurrence Cases

At 4 months after RT, surrounding liver parenchyma hypervascularity during AP (100%) and perfusion defect at the PVP (100%) were observed. At 13 months after RT, tumor size reduction and reduction in tumor vascularity were not observed (Table 3, Figure 3).

### 3.5. Comparison SCEUS Findings with CECT/CEMRI Findings 13 Months after RT

In all 56 no local recurrence cases, at 13 months after RT, tumor size reduction (46/56, 82.1%) and decreased vascularity of the irradiated HCC (56/56, 100%) were observed by SCEUS. Of the 56 no local recurrence cases, 51 cases were available for contrast agents and were evaluated by CECT/CEMRI at 13 months after RT. In seven of the 51 cases, both the irradiated HCC and the surrounding liver parenchyma showed the hypervascularity during AP CECT/CEMRI. This enhancement disturbed the accurate assessment of tumor vascularity and tumor size. In the remaining 44 cases, tumor size reduction (36/44, 81.8%) and decreased vascularity of the irradiated HCC (44/44, 100%) were observed by CECT/CEMRI (Table 2). In all three local recurrence cases, at 13 months after RT, no reduction in tumor size and tumor vascularity was observed by either SCEUS or CECT/CEMRI (Table 3).

### 3.6. Comparison of SCEUS Findings for Cases with and without Local Recurrence 13 Months after RT

At 13 months after RT, when tumor size reduction was observed, the sensitivity, specificity, and accuracy for cases with no local recurrence were 82.1%, 100%, and 83.1%, respectively. At 13 months after RT, when reduction in tumor vascularity was observed, the sensitivity, specificity, and accuracy for cases with no local recurrence were all 100%. At 13 months after RT, tumor size reduction and reduction in tumor vascularity were significantly different between cases with and without local recurrence (*p* = 0.009 and *p* < 0.001, respectively). Hypervascularity of the surrounding liver parenchyma during AP and perfusion defect during the PVP were not significantly different between cases with and without local recurrence 13 months after RT.

### 3.7. Changes in AFP after RT

One case had no blood test data before RT. Of the 58 remaining cases, 36 had newly developed HCC lesions. The remaining 22 cases (19 cases with no local recurrence and 3 local recurrence cases), having high AFP level before RT and no newly developed HCC lesions, were evaluated for changes in AFP level. At 13 months after RT, in 12 of the 19 cases without local recurrence (63.2%), the AFP level had decreased to normal range. The remaining seven cases still had a high AFP level, even though no newly developed HCC lesions were found. In contrast, in two of the three cases with local recurrence (66.7%), the AFP level remained high. However, in the remaining case, the AFP level did not increase and was recorded in the normal range. At 13 months after RFA, when the AFP level of the HCC cases with a high AFP level before RT decreased to normal range, the sensitivity, specificity, and accuracy for cases with no locally recurrent HCC were 63.2%, 66.7%, and 63.6%, respectively.

### 3.8. Additional Treatment for Local Recurrence

All three cases of local HCC recurrence were diagnosed on the basis of imaging examinations at 13 months after RT. After the histological diagnosis of local recurrence of HCCs, these three cases underwent RFA treatment. One case without adverse events experienced local recurrence again and received additional RFA (Figure 3). One had right pleural effusion as an adverse event with RFA; however, no local recurrence was observed during 13 months after RFA. The remaining case had neither adverse events, nor local recurrence, during 14 months after RFA.

### 3.9. Complications

One month after RT, four patients felt fatigue and one patient had a gastric ulcer. No patient required hospitalization. No complications were observed with SCEUS.

## 4. Discussion

Sonazoid has been successfully investigated in many studies, showing high diagnostic accuracy, treatment response assessment, and guidance for HCC [33,34,35,36]. Furthermore, it has been recommended in Japanese guidelines for diagnosing HCC [37]. However, only one study has reported data for CEUS, in terms of its ability to assess the treatment efficacy of RT for HCC. Only Shiozawa et al. have reported CEUS findings after RT using CyberKnife^®^ (Accuray Inc., Sunnyvale, CA, USA) in four cases [38]. Their findings suggested that, if hypervascularity in the liver parenchyma around the HCC is detected in the early period after RT, the HCC may have been adequately irradiated. The reduction in tumor vascularization was considered to indicate the therapeutic efficacy of RT. However, they had a small number of cases, and their description of RT efficacy using CEUS was insufficient, due to the short follow-up periods of less than 1 year [38].

We followed-up 59 patients who underwent RT, obtaining 56 cases with no local recurrence and 3 with local recurrence. Using SCEUS, in the cases with no local recurrence, reduction in tumor vascularity was observed in 87.5% of cases at 4 months, 94.6% at 7 months, 98.2% at 10 months, and 100% at 13 months after RT. Tumor size reduction was observed in 62.5% at 7 months, 73.2% at 10 months, and 82.1% at 13 months after RT. In contrast, in cases with local recurrence, neither a reduction in tumor vascularity nor tumor size was observed even at 13 months after RT. At 13 months after RT, tumor size reduction (*p* = 0.009) and reduction in tumor vascularity (*p* < 0.001) were significantly different between cases with and without local recurrence. Therefore, SCEUS may be useful in evaluating the efficacy of RT for HCC.

Previous studies using CEMRI have reported that tumor enhancement was reduced by 54.3% at 3 months and 74.3% at 6 months after RT [15], while the tumor size was reduced by 66% one year after RT [16]. One of these studies suggested that local recurrence should be defined by growth of >5 mm in size or continuous hyper-enhancement on AP CEMRI during follow-up periods after RT [16]. The results of this report were similar to our findings using SCEUS.

In the present study, even in the cases with no local recurrence, a reduction in tumor vascularity was observed in only 21.4% of cases at one month after RT. In addition, a reduction in tumor size was observed in only 7.1% of cases at one month after RT. It is important to know that even the no local recurrence cases did not show a significant change in US findings at one month after RT. Moreover, follow-up periods of at least 13 months after RT are required, as all three local recurrence cases were found at 13 months after RT. If neither tumor size reduction nor reduction in tumor vascularity are observed at 13 months after RT, the possibility of local recurrence should be considered. In particular, a careful follow-up is necessary for patients who underwent HFRT. Local recurrence cases were observed in 3 of the 59 cases in this study, where two of three local recurrence cases had undergone HFRT. Referring to previous studies, the local control rate is higher in HFRT than in SBRT cases [21,39].

Regarding the hypervascularity of the surrounding liver parenchyma during the AP and the perfusion defect during the PVP SCEUS after RT, findings were observed in cases both with and without local recurrence cases. These findings may indicate the irradiated areas; however, these findings may not help to evaluate the efficacy of RT.

Hypervascularity of the surrounding liver parenchyma around HCC after RT has been mentioned in previous reports [12,40,41,42,43], the results of which are generally consistent with ours. Herfarth et al. reported a change in the image of the liver parenchyma around the tumor, defined as a focal liver reaction (FLR), in contrast-enhanced CT at 1.8 months after RT [40]. In a previous study of CECT, more than 50% of FLR cases showed a hypodensity area in the portal phase 3 to 5 weeks after RT; however, 4 to 5 months later, more than 50% showed a high-density area in the portal phase [40]. This FLR on CECT may correspond to the liver parenchyma hypervascularity during AP SCEUS in our study.

The mechanism of FLR is as follows [12,42,43]: In the irradiated liver, injured endothelial cells undergo apoptosis and Kupffer cells are activated. These injured endothelial cells induce sinusoidal obstruction. The hypoxic environment, due to sinusoidal obstruction, leads to the death of hepatocytes and inflammatory cell infiltration. In addition, Kupffer cells activate hepatic stellate cells. The accumulated hepatic stellate cells lead to liver fibrosis [42,43]. Sinusoidal obstruction and liver fibrosis induce increases in the hepatic arterial flow and decreases in portal blood flow [12]. This increase in the hepatic arterial flow produces enhanced arterial phase contrast effects—namely, hypervascularity—during the arterial phase. The hepatic sinusoidal obstruction causes the concentration of contrast media in dilated sinusoids and the retention of contrast media in the interstitium of hypertrophied fibrous tissue, which results in enhanced portal and late-phase contrast effects of CECT [12]. From one to three months after RT, Kupffer cells show radiation damage [12]. It is thought that the irradiated surrounding liver parenchyma appears as a perfusion defect during the PVP CEUS, which may be due to radiation damage to Kupffer cells and hepatocytes about 4 months after RT treatment. HCC contains few Kupffer cells; thus, it appears as a perfusion defect in the PVP CEUS even before RT. In addition, RT induces HCC necrosis. Therefore, both the irradiated HCC and the surrounding liver parenchyma appear as a perfusion defect during the PVP CEUS from about 4 months after RT.

In this study, 7 of the 19 cases with no local recurrence and a high AFP level before RT showed a high AFP level that persisted 13 months after RT, even in the absence of newly developed HCCs. At 13 months after RT, when the AFP level of HCC cases with high AFP before RT decreased to within normal range, the sensitivity, specificity, and accuracy of cases with no local recurrent HCC were 63.2%, 66.7%, and 63.6%, respectively. Therefore, it may be difficult to determine whether local recurrence has occurred when using AFP. In contrast, at 13 months after RT, when the reduction in tumor vascularity was observed using AP SCEUS, the sensitivity, specificity, and accuracy for cases with no local recurrence were all 100%.

In this study, we used Sonazoid as a contrast agent for CEUS. The re-injection of Sonazoid during the PVP is useful for the detection of wash-in, wash-out HCC, which are ill-defined by grayscale US, and locally recurrent nodules, due to the hypervascularity of the viable HCC [32]. In the present study, tumor vascularity could be evaluated using the re-injection of Sonazoid during the PVP. No patients in this study had complications with SCEUS, although one of the patients experienced an allergy to the CECT contrast agents during follow-up. Our findings demonstrate that SCEUS is a relatively useful and safe modality for evaluating the vascularity of HCC after RT.

There were some limitations to this study: First, the number of cases was small. Only three cases of HCC local recurrence were included. More cases should be added, in order to increase the reliability of the findings of HCC local recurrence and, so, a study with a larger number of cases is needed. Second, this study was a retrospective single-center study. In the future, a prospective multi-center study should be conducted. Third, this study only included lesions that could be assessed by US but, in practice (depending on the location of the tumor and the patient’s body shape), some lesions may not be assessed by US. Fourth, in cases with HCC located near the diaphragm, we used a micro-convex probe to evaluate the tumor vascularity and tumor size, in order to avoid interference by the lung or ribs. The quality of images obtained by the micro-convex probe were slightly inferior to those obtained by the convex probe. Fifth, as this study included five cases with contraindication of CECT/CEMRI, the SCEUS findings after RT were not compared to CECT/CEMRI findings after RT in detail. Sixth, histological proof of the lack of local recurrence of HCCs was not obtained, due to the decrease in size or the impossibility to punctuate for a tumor biopsy.

## 5. Conclusions

Based on this study, SCEUS may be useful in evaluating the efficacy of RT for HCC. SCEUS is simple, repeatable, and non-invasive, especially for patients who are unable to undergo CECT or CEMRI. However, follow-up periods are required for at least 13 months after RT, in order to evaluate the presence of local HCC recurrence.

## Figures and Tables

**Table 1:** Clinical characteristics of enrolled patients.

Baseline Characteristics	All	No Local Recurrence	Local Recurrence
Number of patients/lesions	59/59	56/56	3/3
Age (mean ± SD, years)	72.59 ± 8.768	72.43 ± 8.571	75.67 ± 11.441
Sex (female/male)	19/40	19/37	0/3
Etiology of HCC (hepatitis B/hepatitis C/NBNC/alcohol abuse)	4/46/5/4	4/43/5/4	0/3/0/0
Child-Pugh classification (class A/B)	50/9	47/9	3/0
Diameters of lesion (median, range; mm)	19.6 (8–40)	16.5 (8–40)	20.0 (20–34)
Liver segment (1/2/3/4/5/6/7/8)	6/3/3/12/1/0/11/23	6/2/3/11/1/0/11/22	0/1/0/1/0/0/0/1
RT (SBRT/HFRT)	48/11	47/9	1/2
Follow-up period ^a^ (median (range) in months)	44.5 (13–82)	48 (16–82)	21 (20–22)

NBNC, non-B, non-C hepatitis; RT, radiation therapy; HFRT, hypofractionated radiotherapy; SBRT, stereotactic body radiation therapy. ^a^ Follow-up every three months was continued as long as the patient was alive and could visit the hospital.

**Table 2:** Changes in SCEUS (N = 56) and CECT/CEMRI (N = 51) findings during 13 months after RT for no local recurrence cases.

Observed Parameters	Follow-Up Period Number (%)									
	1 Month		4 Months		7 Months		10 Months		13 Months	
Modality	US	C/M	US	C/M ^e^	US	C/M ^e^	US	C/M ^f^	US	C/M ^f^
Tumor size										
Reduction ^a^	4 (7.1)	9 (17.6)	17 (30.4)	15 (33.3)	35 (62.5)	29 (64.4)	41 (73.2)	37 (84.1)	46 (82.1)	37 (84.1)
Increase ^b^	0 (0)	0 (0)	0 (0)	0 (0)	0 (0)	0 (0)	0 (0)	0 (0)	0 (0)	0 (0)
No change ^c^	52 (92.9)	42 (82.4)	39 (69.6)	30 (66.7)	21 (37.5)	16 (35.6)	15 (26.8)	7 (15.9)	10 (17.9)	7 (15.9)
Tumor vascularity										
Reduction ^d^	12 (21.4)	9 (17.6)	49 (87.5)	30 (66.7)	53 (94.6)	37 (82.2)	55 (98.2)	42 (95.5)	56 (100)	44 (100)
No change	44 (78.6)	42 (82.4)	7 (12.5)	15 (33.3)	3 (5.4)	8 (17.8)	1 (1.8)	2 (4.5)	0 (0)	0 (0)
Hypervascularity of surrounding liver parenchyma during AP										
Presence	22 (39.3)		50 (89.3)		56 (100)		56 (100)		51 (91.1)	
Absence	34 (60.7)		6 (10.7)		0 (0)		0 (0)		5 (8.9)	
Perfusion defect during PVP										
Presence	8 (14.3)		49 (87.5)		54 (96.4)		56 (100)		56 (100)	
Absence	48 (85.7)		7 (12.5)		2 (3.6)		0 (0)		0 (0)	

SCEUS, contrast-enhanced ultrasound using Sonazoid; CECT/CEMRI, contrast-enhanced CT/contrast-enhanced MRI; US, contrast-enhanced ultrasound using Sonazoid; C/M, contrast-enhanced CT/contrast-enhanced MRI; AP, arterial phase; PVP, post-vascular phase; ^a^ Reduction of more than 30%, compared to the maximum diameter of the HCC lesion measured before treatment. ^b^ Increase of more than 20%, compared to the maximum diameter of the HCC lesion measured before treatment. ^c^ Neither sufficient reduction to qualify for “Reduction” nor sufficient increase to qualify for “Increase”. ^d^ Reduction of tumor vascularity, compared to the pre-treatment vascularity, using AP or re-injection during PVP contrast-enhanced ultrasound using Sonazoid. ^e^ In six of the 51 cases, both the irradiated HCC and the surrounding liver parenchyma showed the hypervascularity during AP CECT/CEMRI, which interfering with the accurate assessment of tumor vascularity and tumor size. In the remaining 45 cases, tumor size and tumor vascularity were evaluated by CECT/CEMRI. ^f^ In seven of the 51 cases, both the irradiated HCC and the surrounding liver parenchyma showed the hypervascularity during AP CECT/CEMRI, which interfering with the accurate assessment of tumor vascularity and tumor size. In the remaining 44 cases, tumor size and tumor vascularity were evaluated by CECT/CEMRI.

**Table 3:** Changes in SCEUS and CECT/CEMRI findings during 13 months after RT for local recurrence cases (N = 3).

Observed Parameters	Follow-Up Period Number (%)									
	1 Month		4 Months		7 Months		10 Months		13 Months	
**Modality**	US	C/M	US	C/M	US	C/M	US	C/M	US	C/M
Tumor size										
Reduction ^a^	0 (0)	0 (0)	0 (0)	0 (0)	0 (0)	0 (0)	0 (0)	0 (0)	0 (0)	0 (0)
Increase ^b^	0 (0)	0 (0)	0 (0)	0 (0)	0 (0)	0 (0)	0 (0)	0 (0)	0 (0)	0 (0)
No change ^c^	3 (100)	3 (100)	3 (100)	3 (100)	3 (100)	3 (100)	3 (100)	3 (100)	3 (100)	3 (100)
Tumor vascularity										
Reduction ^d^	0 (0)	0 (0)	0 (0)	0 (0)	0 (0)	0 (0)	0 (0)	0 (0)	0 (0)	0 (0)
No change	3 (100)	3 (100)	3 (100)	3 (100)	3 (100)	3 (100)	3 (100)	3 (100)	3 (100)	3 (100)
Hypervascularity of surrounding liver parenchyma during AP										
Presence	0 (0)		3 (100)		3 (100)		3 (100)		3 (100)	
Absence	3 (100)		0 (0)		0 (0)		0 (0)		0 (0)	
Perfusion defect during PVP										
Presence	0 (0)		3 (100)		3 (100)		3 (100)		3 (100)	
Absence	3 (100)		0 (0)		0 (0)		0 (0)		0 (0)	

SCEUS, contrast-enhanced ultrasound using Sonazoid; CECT/CEMRI, contrast-enhanced CT/contrast-enhanced MRI; US, contrast-enhanced ultrasound using Sonazoid; C/M, contrast-enhanced CT/contrast-enhanced MRI; AP, arterial phase; PVP, post-vascular phase; ^a^ Reduction of more than 30%, compared to the maximum diameter of the HCC lesion measured before treatment. ^b^ Increase of more than 20%, compared to the maximum diameter of the HCC lesion measured before treatment. ^c^ Neither sufficient reduction to qualify for “Reduction” nor sufficient increase to qualify for “Increase”. ^d^ Reduction of tumor vascularity, compared to the pre-treatment vascularity, using AP or re-injection during PVP contrast-enhanced ultrasound using Sonazoid.

## Data Availability

Not applicable.

## References

[1] Bray F., Ferlay J., Soerjomataram I., Siegel R.L., Torre L.A., Jemal A. (2018). Global Cancer Statistics 2018: GLOBOCAN Estimates of Incidence and Mortality Worldwide for 36 Cancers in 185 Countries. Cancer J. Clin..

[2] Villanueva A. (2019). Hepatocellular Carcinoma. N. Engl. J. Med..

[3] Siegel R.L., Miller K.D., Jemal A. (2020). Cancer statistics, 2020. CA Cancer J. Clin..

[4] Forner A., Reig M.E., de Lope C.R., Bruix J. (2010). Current strategy for staging and treatment: The BCLC update and future prospects. Semin. Liver Dis..

[5] Ayuso C., Rimola J., Vilana R., Burrel M., Darnell A., Garcia-Criado A., Bianchi L., Belmonte E., Caparroz C., Barrufet M. (2018). Diagnosis and staging of hepatocellular carcinoma (HCC): Current guidelines. Eur. J. Radiol..

[6] Nault J.C., Sutter O., Nahon P., Ganne-Carrie N., Seror O. (2018). Percutaneous treatment of hepatocellular carcinoma: State of the art and innovations. J. Hepatol..

[7] Kang J.K., Kim M., Cho C.K., Yang K.M., Yoo H.J., Kim J.H., Bae S.H., Jung D.H., Kim K.B., Lee D.H. (2012). Stereotactic body radiation therapy for inoperable hepatocellular carcinoma as a local salvage treatment after incomplete transarterial chemoembolization. Cancer.

[8] Bujold A., Massey C.A., Kim J.J., Brierley J., Cho C., Wong R.K., Dinniwell R.E., Kassam Z., Ringash J., Cummings B. (2013). Sequential phase I and II trials of stereotactic body radiotherapy for locally advanced hepatocellular carcinoma. J. Clin. Oncol..

[9] Moore A., Cohen-Naftaly M., Tobar A., Kundel Y., Benjaminov O., Braun M., Issachar A., Mor E., Sarfaty M., Bragilovski D. (2017). Stereotactic body radiation therapy (SBRT) for definitive treatment and as a bridge to liver transplantation in early stage inoperable Hepatocellular carcinoma. Radiat. Oncol..

[10] Hara K., Takeda A., Tsurugai Y., Saigusa Y., Sanuki N., Eriguchi T., Maeda S., Tanaka K., Numata K. (2019). Radiotherapy for Hepatocellular Carcinoma Results in Comparable Survival to Radiofrequency Ablation: A Propensity Score Analysis. Hepatology.

[11] Kim N., Cheng J., Jung I., Liang J.D., Shih Y.L., Huang W., Kimura T., Lee V.H.F., Zeng Z.C., Zhenggan R. (2020). Stereotactic body radiation therapy vs. radiofrequency ablation in Asian patients with hepatocellular carcinoma. J. Hepatol..

[12] Takamatsu S., Kozaka K., Kobayashi S., Yoneda N., Yoshida K., Inoue D., Kitao A., Ogi T., Minami T., Kouda W. (2018). Pathology and images of radiation-induced hepatitis: A review article. Jpn. J. Radiol..

[13] Kimura T., Takahashi S., Takahashi I., Nishibuchi I., Doi Y., Kenjo M., Murakami Y., Honda Y., Aikata H., Chayama K. (2015). The Time Course of Dynamic Computed Tomographic Appearance of Radiation Injury to the Cirrhotic Liver Following Stereotactic Body Radiation Therapy for Hepatocellular Carcinoma. PLoS ONE.

[14] Mendiratta-Lala M., Gu E., Owen D., Cuneo K.C., Bazzi L., Lawrence T.S., Hussain H.K., Davenport M.S. (2018). Imaging Findings Within the First 12 Months of Hepatocellular Carcinoma Treated With Stereotactic Body Radiation Therapy. Int. J. Radiat. Oncol. Biol. Phys..

[15] Oldrini G., Huertas A., Renard-Oldrini S., Taste-George H., Vogin G., Laurent V., Salleron J., Henrot P. (2017). Tumor response assessment by MRI following stereotactic body radiation therapy for hepatocellular carcinoma. PLoS ONE.

[16] Mendiratta-Lala M., Masch W., Shankar P.R., Hartman H.E., Davenport M.S., Schipper M.S., Maurino C., Cuneo K.C., Lawrence T.S., Owen D. (2019). Magnetic Resonance Imaging Evaluation of Hepatocellular Carcinoma Treated With Stereotactic Body Radiation Therapy: Long Term Imaging Follow-Up. Int. J. Radiat. Oncol. Biol. Phys..

[17] Omata M., Cheng A., Kokudo N., Kudo M., Lee J.M., Jia J., Tateishi R., Han K.H., Chawla Y.K., Shiina S. (2017). Asia-Pacific clinical guidelines on the management of hepatocellular carcinoma: 2017 update. Hepatol. Int..

[18] Heimbach J.K., Kulik L.M., Finn R.S., Sirlin C.B., Abecassis M.M., Roberts L.R., Zhu A.X., Murad M.H., Marrero J.A. (2018). AASLD Guidelines for the Treatment of Hepatocellular Carcinoma. Hepatology.

[19] European Association for the Study of the Liver (2018). EASL Clinical Practice Guidelines: Management of hepatocellular carcinoma. J. Hepatol..

[20] Lencioni R., Llovet J.M. (2010). Modified RECIST (mRECIST) Assessment for Hepatocellular Carcinoma. Semin. Liver Dis..

[21] Kibe Y., Takeda A., Tsurugai Y., Eriguchi T. (2020). Local control by salvage stereotactic body radiotherapy for recurrent/residual hepatocellular carcinoma after other local therapies. Acta Oncol..

[22] Mori Y., Hayakawa A., Abe K., Tanigawa M., Takahashi M., Naruse H., Yamaguchi H. (2011). The safety and the efficacy of ultrasound contrast media Perflubutane microbubbles in clinical practice. Choonpa Igaku Jpn. Soc. Ultrason. Med..

[23] Claudon M., Dietrich C.F., Choi B.I., Cosgrove D.O., Kudo M., Nolsøe C.P., Piscaglia F., Wilson S.R., Barr R.G., Chammas M.C. (2013). Guidelines and good clinical practice recommendations for contrast enhanced ultrasound (CEUS) in the liver-update 2012: A WFUMB-EFSUMB initiative in cooperation with representatives of AFSUMB, AIUM, ASUM, FLAUS and ICUS. Ultrason. Med. Biol..

[24] Westwood M., Joore M., Grutters J., Redekop K., Armstrong N., Lee K., Gloy V., Raatz H., Misso K., Severens J. (2013). Contrast enhanced ultrasound using SonoVue^®^ (sulphur hexafluoride microbubbles) compared with contrast-enhanced computed tomography and contrastenhanced magnetic resonance imaging for the characterisation of focal liver lesions and detection of liver metastases: A systematic review and cost-effectiveness analysis. Health Technol. Assess..

[25] Wiesinger I., Wiggermann P., Zausig N., Beyer L.P., Salzberger B., Stroszczynski C., Jung E.M. (2018). Percutaneous treatment of malignant liver lesions: Evaluation of success using contrast- enhanced ultrasound (CEUS) and perfusion software. Ultraschall Med..

[26] Nishigori S., Numata K., Irie K., Fukuda H., Chuma M., Maeda S. (2018). Fusion imaging with contrast-enhanced ultrasonography for evaluating the early therapeutic efficacy of radiofrequency ablation for small hypervascular hepatocellular carcinomas with iso-echoic or unclear margins on conventional ultrasonography. J. Med. Ultrason..

[27] Numata K., Fukuda H., Morimoto M., Kondo M., Nozaki A., Oshima T., Okada M., Takebayashi S., Maeda S., Tanaka K. (2012). Use of fusion imaging combining contrast-enhanced ultrasonography with a perflubutane-based contrast agent and contrast-enhanced computed tomography for the evaluation of percutaneous radiofrequency ablation of hypervascular hepatocellular carcinoma. Eur. J. Radiol..

[28] Sugimori K., Numata K., Okada M., Nihonmatsu H., Takebayashi S., Maeda S., Nakano M., Tanaka K. (2017). Central vascular structures as a characteristic finding of regenerative nodules using hepatobiliary phase gadolinium ethoxybenzyl diethylenetriaminepentaacetic acid-enhanced MRI and arterial dominant phase contrastenhanced US. J. Med. Ultrason..

[29] Wang F., Numata K., Nihonmatsu H., Okada M., Maeda S. (2020). Application of new ultrasound techniques for focal liver lesions. J. Med. Ultrason..

[30] Duisyenbi Z., Numata K., Nihonmatsu H., Fukuda H., Chuma M., Kondo M., Nozaki A., Tanaka K., Maeda S. (2019). Comparison between low mechanical index and high mechanical index contrast modes of Contrast-Enhanced ultrasonography: Evaluation of perfusion defects of hypervascular hepatocellular carcinomas during the Post-Vascular phase. J. Ultrasound Med..

[31] Takeda A., Sanuki N., Tsurugai Y., Iwabuchi S., Matsunaga K., Ebinuma H., Imajo K., Aoki Y., Saito H., Kunieda E. (2016). Phase 2 study of stereotactic body radiotherapy and optional transarterial chemoembolization for solitary hepatocellular carcinoma not amenable to resection and radiofrequency ablation. Cancer.

[32] Kudo M., Hatanaka K., Maekawa K. (2010). Newly developed Novel Ultrasound Technique, Defect Reperfusion Ultrasound Imaging, Using Sonazoid in the management of hepatocellular carcinoma. Oncology.

[33] Numata K., Morimoto M., Ogura T., Sugimori K., Takebayashi S., Okada M., Tanaka K. (2008). Ablation Therapy Guided by Contrast-Enhanced Sonography with Sonazoid for Hepatocellular Carcinoma Lesions Not Detected by Conventional Sonography. J. Ultrasound Med..

[34] Sugimoto K., Moriyasu F., Saito K., Taira J., Saguchi T., Yoshimura N., Oshiro H., Imai Y., Shiraishi J. (2012). Comparison of Kuppfer-Phase Sonazoid-Enhanced Sonography and Hepatobiliary-Phase Gadoxetic Acid-Enhanced Magnetic Resonance Imaging of Hepatocellular Carcinoma and Correlation With Histologic Grading. J. Ultrasound Med..

[35] Inoue T., Hyodo T., Korenaga K., Murakami T., Imai Y., Higashi A., Suda T., Takano T., Miyoshi K., Koda M. (2016). Kuppfer phase image of Sonazoid-enhanced US is useful in predicting a hypervascularization of non-hypervascular hypointense hepatic lesions detected on Gd-EOB-DTPA-enhanced MRI: A multicenter retrospective study. J. Gastroenterol..

[36] Wang F., Numata K., Nakano M., Tanabe M., Chuma M., Nihonmatsu H., Nozaki A., Ogushi K., Luo W., Ruan L. (2020). Diagnostic Value of Imaging Methods in the Histological Four Grading of Hepatocellular Carcinoma. Diagnostics.

[37] Kudo M., Matsui O., Izumi N., Iijima H., Kadoya M., Imai Y., Okusaka T., Miyayama S., Tsuchiya K., Ueshima K. (2014). JSH Concensus-Based Clinical Practice Guidelines for the Management of Hepatocellular Carcinoma: 2014 Update by the Liver Cancer Study Group of Japan. Liver Cancer.

[38] Shiozawa K., Watanabe M., Ikehara T., Kobayashi K., Ochi Y., Suzuki Y., Fuchinoue K., Yoneda M., Kenmochi T., Okubo Y. (2016). Evaluation of contrast-enhanced ultrasonography for hepatocellular carcinoma prior to and following stereotactic body radiation therapy using the CyberKnife system: A preliminary report. Oncol. Lett..

[39] Bae S.H., Park H.C., Lim D.H., Lee J.A., Gwak G.Y., Choi M.S., Lee J.H., Koh K.C., Palik S.W., Yoo B.C. (2012). Salvage Treatment With Hypofractionated Radiotherapy in Patients With Recurrent Small Hepatocellular Carcinoma. Int. J. Radiat. Oncol. Biol. Phys..

[40] Herfarth K.K., Hof H., Bahner M.L., Lohr F., Hoss A., van Kaick G., Wannenmacher M., Debus J. (2003). Assessment of focal liver reaction by multiphasic CT after stereotactic single-dose radiotherapy of liver tumors. Int. J. Radiat. Oncol. Biol. Phys..

[41] Sanuki-Fujimoto N., Takeda A., Ohashi T., Kunieda E., Iwabuchi S., Takatsuka K., Koike N., Shigematsu N. (2010). CT evaluations of focal liver reactions following stereotactic body radiotherapy for small hepatocellular carcinoma with cirrhosis: Relationship between imaging appearance and baseline liver function. Br. J. Radiol..

[42] Kim J., Jung Y. (2017). Radiation-induced liver disease: Current understanding and future perspectives. Exp. Mol. Med..

[43] Deleve L.D., Shulman H.M., McDonald G.B. (2002). Toxic injury to hepatic sinusoids: Sinusoidal obstruction syndrome (veno-occlusive disease). Semin. Liver Dis..

